# Comprehensive characterisation of individuals with fibrotic interstitial lung disease: baseline insights from the INJUSTIS study

**DOI:** 10.1136/bmjresp-2024-003112

**Published:** 2026-01-20

**Authors:** Fasihul Khan, Iain Stewart, Lucy Howard, Christopher Michael Barber, Rebecca Borton, Rebecca Braybrooke, Glenn Hearson, Steve Jones, Toby Maher, Laura Matthews, Gauri Saini, Norma Thompson, Andrew M Wilson, Simon R Johnson, Gisli Jenkins

**Affiliations:** 1 Respiratory Medicine, University of Nottingham, Nottingham, UK; 2 Respiratory Medicine, University Hospitals of Leicester NHS Trust, Leicester, UK; 3 National Heart & Lung Institute, Imperial College London, London, UK; 4 Adelphi Real World, Bollington, UK; 5 Respiratory Medicine, Sheffield Teaching Hospitals NHS Foundation Trust, Sheffield, UK; 6 Patientmpower Ltd, Dublin, UK; 7 Action for Pulmonary Fibrosis, Peterborough, UK; 8 USC Keck School of Medicine, Los Angeles, California, USA; 9 Respiratory Medicine, Nottingham Respiratory Research Unit, Nottingham, UK; 10 Norwich Medical School, University of East Anglia, Norwich, UK; 11 National Heart and Lung Institute, Imperial College London, London, UK

**Keywords:** Idiopathic Pulmonary Fibrosis, Interstitial Fibrosis

## Abstract

**Background:**

Interstitial lung disease (ILD) represents a group of complex parenchymal conditions characterised by varying clinical trajectories. The It’s Not JUST Idiopathic Pulmonary Fibrosis Study seeks to identify genetic, proteomic and clinical biomarkers that distinguish rapidly progressive fibrotic phenotypes from stable phenotypes irrespective of aetiology. This manuscript presents baseline insights from the recruited cohort.

**Methods:**

In this prospective, longitudinal study, participants with fibrotic ILDs, including idiopathic pulmonary fibrosis (IPF), fibrotic hypersensitivity pneumonitis, rheumatoid arthritis-associated ILD, asbestosis and unclassifiable ILD, were enrolled from 24 UK sites. Participants underwent comprehensive baseline evaluation including demographics, exposure history, lung function testing, 6-min walk tests, blood sampling and standardised questionnaires to assess symptoms and quality of life.

**Results:**

A total of 272 participants were recruited, predominantly older white males with a smoking history. Baseline lung function showed comparable forced vital capacity (mean 89.0% predicted), diffusion of carbon monoxide (mean 57.9% predicted) and 6-min walk distance (mean 302 m) across ILD subtypes. Hypertension was the most prevalent comorbidity, affecting 40.8% of participants, with no significant differences across subtypes. Anxiety and depression were notably lower in IPF than non-IPF (4.5%; 21.0%). Previous occupational exposure was reported in 68.8% of participants, with asbestos exposure the most prevalent (36%). Bird exposure was reported by 40.4% of participants, with no significant differences across subtypes. No significant differences in health-related quality of life scores were observed across subtypes.

**Conclusions:**

Despite varied aetiologies, fibrotic ILDs exhibit demographic and functional similarities, including lung function and health-related quality of life suggesting commonalities in disease mechanisms.

**Trial registration number:**

NCT03670576.

**WHAT IS ALREADY KNOWN ON THIS TOPIC**

- Fibrotic interstitial lung diseases (ILDs) are characterised by progressive fibrosis, with limited understanding of risk factors associated with poor outcomes.

**WHAT THIS STUDY ADDS**

- This study offers a detailed baseline analysis of demographic, clinical, comorbidity and exposure profiles in patients with fibrotic ILD. These insights enhance our understanding of the common and distinct risk profiles associated with fibrotic ILDs.

**HOW THIS STUDY MIGHT AFFECT RESEARCH, PRACTICE OR POLICY**

- The findings lay a strong foundation for future research integrating advanced genomic, transcriptomic and proteomic analyses to advance personalised treatment strategies in progressive pulmonary fibrosis.

## Introduction

 Interstitial lung disease (ILD) encompasses a heterogeneous group of inflammatory and fibrotic conditions of the lung parenchyma associated with substantial morbidity and mortality.^1^ Idiopathic pulmonary fibrosis (IPF) is often considered the archetypal progressive fibrotic ILD, yet a significant proportion of patients with other fibrotic ILDs including fibrotic hypersensitivity pneumonitis (fHP), rheumatoid arthritis-related ILD (RA-ILD) and unclassifiable ILD (uILD) demonstrate progressive disease behaviour and respond to antifibrotic therapies,^2^,^4^ suggesting potential common mechanistic pathways regardless of aetiology.

International consensus statements and expert perspectives have proposed criteria to define progressive pulmonary fibrosis (PPF) utilising a combination of clinical, physiological and radiological parameters.^1 5^ While these criteria are effective in identifying patients at increased risk of poor outcomes, crucially they primarily rely on the manifestation of irreversible fibrosis and lung function decline, rather than enabling the early identification of patients prior to disease progression. The recent extension of antifibrotic therapies to encompass PPF has generated an urgent need to identify novel biomarkers that can predict disease behaviour, facilitate early prognostication and guide therapy stratification, thereby enabling personalised medicine. Furthermore, such biomarkers have the potential to enhance our understanding of common disease pathways across fibrotic ILDs and inform future drug development. Although blood-based biomarkers reflecting various mechanistic pathways have been described in IPF,^6 7^ there remains a significant gap in our understanding of biomarkers applicable to other fibrotic ILDs.

The It’s Not JUST Idiopathic Pulmonary Fibrosis Study (INJUSTIS) is an on-going prospective, longitudinal cohort study across 24 sites in the UK involving incident diagnoses of IPF, fHP, RA-ILD, asbestosis and uILD.^8^ The study aims to explore and identify genetic, blood-based and clinical biomarkers that may distinguish distinct phenotypes of progressive fibrotic phenotypes irrespective of aetiology and traditional clinical, radiological and pathological phenotyping. We hypothesise that certain biomarkers and pathways are common to progressive fibrotic ILDs. Herein, the baseline demographic and medical characteristics of participants enrolled in INJUSTIS are presented, providing crucial insights and laying the foundation for subsequent analyses exploring predictive biomarkers and therapeutic targets.

## Methods

### Study design and participants

The comprehensive design and methodology for the INJUSTIS study have been previously described.^8^ The study enrolled participants aged 18 and older with multidisciplinary-confirmed diagnoses of fibrotic ILD, evidenced by reticulation and traction airway dilatation attributed to fHP, RA-ILD, asbestosis or uILD. Recruitment occurred at outpatient clinics across 24 UK sites. All participants with fibrotic ILD, irrespective of their progression status, were eligible for inclusion. Exclusions comprised individuals with inflammatory radiological changes lacking fibrosis, or those diagnosed more than 18 months before study commencement. RA-ILD was selected due to its established enriched association with a fibrotic, progressive phenotype compared with other connective tissue disease (CTD)-ILDs.^9^ Efforts were made to maintain consistency in recruitment numbers for each of the ILD subtypes to allow for meaningful comparisons. Additionally, a reference cohort comprising participants with IPF, diagnosed in accordance with internationally accepted criteria,^1 10^ was recruited. All patients presenting at outpatient clinics who met the study’s inclusion and exclusion criteria were approached for participation. The study was conducted in accordance with the protocol,^8^ and all participants provided written informed consent. The study was registered on clinicaltrials.gov (NCT03670576).

### Participant history and procedures

Following enrolment and written informed consent, participants underwent a baseline visit where demographic characteristics and relevant medical information including comorbidities, medications, occupation and exposure history were recorded (online supplemental material). Blood samples were obtained for routine laboratory tests including a full blood count, with additional samples collected and processed, according to standardised operating procedures and stored at −80ºC prior to analysis. Antibody results, along with liver function tests and bone profile, were gathered from historical records. Physiological investigations, including spirometry, gas transfer and 6-min walk tests, were performed according to the American Thoracic Society (ATS)/European Respiratory Society (ERS) standards.^11 12^ DL_CO_ and FVC percent predicted values were calculated using Global Lung Initiative equations.^13 14^ Significant asbestos exposure was defined as 1 year of heavy exposure or 5–10 years of moderate exposure.

Patient-reported outcome measures were collected using standardised questionnaires. The Leicester Cough Questionnaire was used to evaluate cough impact, encompassing physical, psychological and social domains, with higher scores indicating reduced cough-related symptoms and improved quality of life.^15^ The Medical Research Council (MRC) dyspnoea scale was used to assess breathlessness severity based on activity level, up to a maximum score of 5, with higher scores indicating increased exertional breathlessness.^16^ IPF Prognostic Assessment and Referral to Care (IPARC) evaluated distress of IPF symptoms and their impact on daily activities, yielding a maximum score of 33, with higher scores indicative of greater symptom severity.^17^ King’s Brief ILD Questionnaire (KBILD) comprised breathlessness, activities and psychological domains, providing an overall score ranging from 0 to 100, with higher scores denoting better health-related quality of life (HRQOL).^18^ EuroQol 5-dimension 5-level(EQ-5D-5L) measured holistic health across physical, psychological and social domains, with an index score ranging up to 1, and a quantitative self-perceived health score ranging from 0 to 100, with 0 representing the worst imaginable health state and 100 representing the best.^19^

### Data management and analyses

Locally generated data were entered into an encoded electronic case report form and stored (medrio EDC) under anonymous identifiers. Internal consistency checks were performed, and data were verified against source documents. The baseline database was locked on 29 January 2024, and statistical analyses were conducted using Stata V.18.0. Descriptive analyses were conducted to characterise the study population and provide an overview of baseline demographics and clinical characteristics, presented in tabular format, enabling comparisons between ILD subtypes. Categorical variables were summarised as frequencies and percentages, while continuous variables were described using means and SD or medians and IQRs. Group differences were analysed using independent t-tests or one-way analysis of variance for normally distributed continuous variables, Wilcoxon rank sum or Kruskal-Wallis test for non-normally distributed continuous variables and χ^2^ or Fisher’s exact tests for categorical variables. A significance level of p <0.05 was considered statistically significant. Missing data were handled by excluding cases with incomplete information from the respective analyses.

### Patient and public involvement

Patients were actively involved in the design of the study and contributed to shaping its objectives and methodology. Patient representatives also reviewed and provided feedback on all patient-facing materials to ensure they were accessible, appropriate and aligned with patient priorities. A steering committee including an independent chairperson, clinicians, researchers and patient representatives was established and convened regularly.

## Results

A total of 272 participants with a confirmed diagnosis of fibrotic ILD were recruited between November 2018 and May 2023, distributed across five diagnostic subtypes: IPF (n=67), asbestosis (n=54), fibrotic HP (n=54), RA-ILD (n=47) and uILD (n=50). The recruitment period was extended due to the COVID-19 pandemic, which adversely impacted patient recruitment. Although completion of recruitment to the IPF cohort preceded that of other subtypes, enrolment persisted across all subtypes until the predetermined sample size for each ILD subtype was attained to mitigate potential time-related bias. The median time from diagnosis to recruitment was 5.8 months (IQR 1.9–14) in the entire cohort, with no significant difference in time from diagnosis to enrolment across the ILD subtypes (p=0.17).

### Clinical and demographic characteristics

All participants received a confirmed diagnosis through multidisciplinary team assessment, with surgical lung biopsy performed in 10 (3.7%) cases. Cryobiopsies were not performed, and bronchoalveolar lavage was carried out in 11 participants as part of an optional substudy. The IPF and non-IPF cohorts were predominantly male (76.1% IPF, 66.8% non-IPF), of white ethnicity (100% IPF, 93.7% non-IPF), with a smoking history (62.1% IPF, 68.3% non-IPF) and mean ages of 72.3±7.8 and 71.0±9.4 in IPF and non-IPF, respectively (table 1). Participants with asbestosis were older than other ILD subtypes on average (75.9 years ±6.3) and had the greatest proportion of male participants (98.1%). A positive family history of ILD in first-degree or second-degree relatives was reported in 15.2% (IPF) and 10.2% (non-IPF) of participants. Baseline percent predicted forced vital capacity (FVC) and gas transfer for carbon monoxide (DL_CO_) was comparable between IPF (FVC 90.6±19.8, DL_CO_ 58.3±15.5) and non-IPF (FVC 88.5±21.5, DL_CO_ 57.7±17.9). Participants with fibrotic HP had the greatest impairment in percent predicted lung function (FVC 80.0±21.1, DL_CO_ 53.5±17.0). The mean (SD) 6-min walk distance was 311 m±132 (IPF) and 299±116 (non-IPF), with notably reduced exercise tolerance observed in participants with asbestosis (276 m±114 m). The median ILD-gender-age-physiology(GAP) score was 3 in both IPF (IQR 3–4) and non-IPF (IQR 2–4) groups. Across specific ILD subtypes, median scores were consistent at 3, with a slightly higher lower bound (IQ 3–4) observed in asbestosis. Body mass index was 29.0±4.9 in IPF participants and 29·2±5.6 in non-IPF participants, showing little difference across the ILD subtypes.

**Table 1:** Baseline demographics and clinical characteristics of included participants

	All (n=272)	IPF (n=67)	Non-IPF (n=205)	Asbestosis (n=54)	Fibrotic HP (n=54)	RA-ILD (n=47)	uILD (n=50)	P value
Age, years	71.3 (9.0)	72.3 (7.8)	71.0 (9.4)	75.9 (6.3)	68.6 (9.2)	71.0 (8.1)	68.0 (11.3)	<0.001
Sex								
Male	188 (69.1%)	51 (76.1%)	137 (66.8%)	53 (98.1%)	30 (55.6%)	22 (46.8%)	32 (64.0%)	<0.001
Female	84 (30.1%)	16 (23.9%)	68 (33.2%)	1 (1.9%)	24 (44.4%)	25 (52.2%)	18 (36.0%)	
Ethnicity								
White	259 (95.2%)	67 (100%)	192 (93.7%)	54 (100%)	49 (90.7%)	43 (91.5%)	46 (92.0%)	0.003
Black	3 (1.1%)	0	3 (1.5%)	0	0	1 (2.1%)	2 (4.0%)	
Asian	6 (2.2%)	0	6 (2.9%)	0	5 (9.3%)	1 (2.1%)	0	
Other	4 (1.5%)	0	4 (2.0%)	0	0	2 (4.3%)	2 (4.0%)	
Smoking								
Current/ex	181 (66.8%)	41 (62.1%)	140 (68.3%)	41 (75.9%)	31 (57.4%)	34 (72.3%)	34 (68.0%)	0.02
Never	90 (33.2%)	25 (37.9%)	65 (31.7%)	13 (24.1%)	23 (42.6%)	13 (27.7%)	16 (32.0%)	
Pack-years	19.5 (8-30)	19 (9–33)	19.7 (7.8–30)	18.6 (10-27)	10.5 (4.8–32)	20 (8–36)	20 (5.8–30)	0.88
BMI (kg/m^2^)	29.1 (5.4)	29.0 (4.9)	29.2 (5.6)	28.8 (4.7)	29.5 (6.7)	27.8 (3.9)	30.6 (6.3)	0.15
Family history of ILD	31 (11.4%)	10 (15.2%)	21 (10.2%)	4 (7.4%)	7 (13.0%)	3 (6.4%)	7 (14.0%)	0.49
Lung physiology								
FEV1 (% predicted)	86.8 (23)	87.6 (21.6)	86.6 (23.5)	87.9 (19.8)	80.1 (25.5)	92 (21.6)	87.3 (25.6)	0.12
FVC, L	2.85 (0.86)	3.00 (0.89)	2.80 (0.84)	3.08 (0.71)	2.55 (0.92)	2.81 (0.86)	2.78 (0.80)	0.01
FVC (% predicted)	89.0 (21.1)	90.6 (19.8)	88.5 (21.5)	93.7 (19.5)	80.0 (21.1)	93.4 (18.5)	87.3 (24.3)	0.01
DL_CO_ (% predicted)	57.9 (17.3)	58.3 (15.5)	57.7 (17.9)	59.4 (18.1)	53.5 (17.0)	60.0 (17.9)	58.2 (18.4)	0.39
GAP score	3 (2-4)	3 (3-4)	3 (2-4)	3 (3-4)	3 (2-4)	3 (2-4)	3 (2-4)	0.02
6MWD (m)	302 (120)	311 (132)	299 (116)	276 (114)	307 (116)	298 (120)	315 (114)	0.49
Time from diagnosis to enrolment (months)	5.8 (1.9–14)	8.8 (3.4–16.4)	5.2 (1.8–13.0)	4.7 (2.2–10.0)	7.4 (1.9–18.0)	7.5 (1.9–14.3)	4.9 (1.3–9.9)	0.17

Data are presented as percentages, mean and SD or medians and IQR. Family history defined as history of ILD in a first or second-degree relative.

P values indicate the significance of differences between ILD subtypes (IPF, asbestosis, fibrotic HP, RA-ILD, unclassifiable ILD).

BMI, body mass index; DL_CO_, gas transfer for carbon monoxide; FEV1, forced expiratory volume in 1 s; FVC, forced vital capacity; HP, hypersensitivity pneumonitis; ILD, interstitial lung disease; IPF, idiopathic pulmonary fibrosis; 6MWD, 60-min walk distance; RA-ILD, rheumatoid arthritis ILD; uILD, unclassifiable ILD.

### Comorbidities and medications

**Figure 1:**
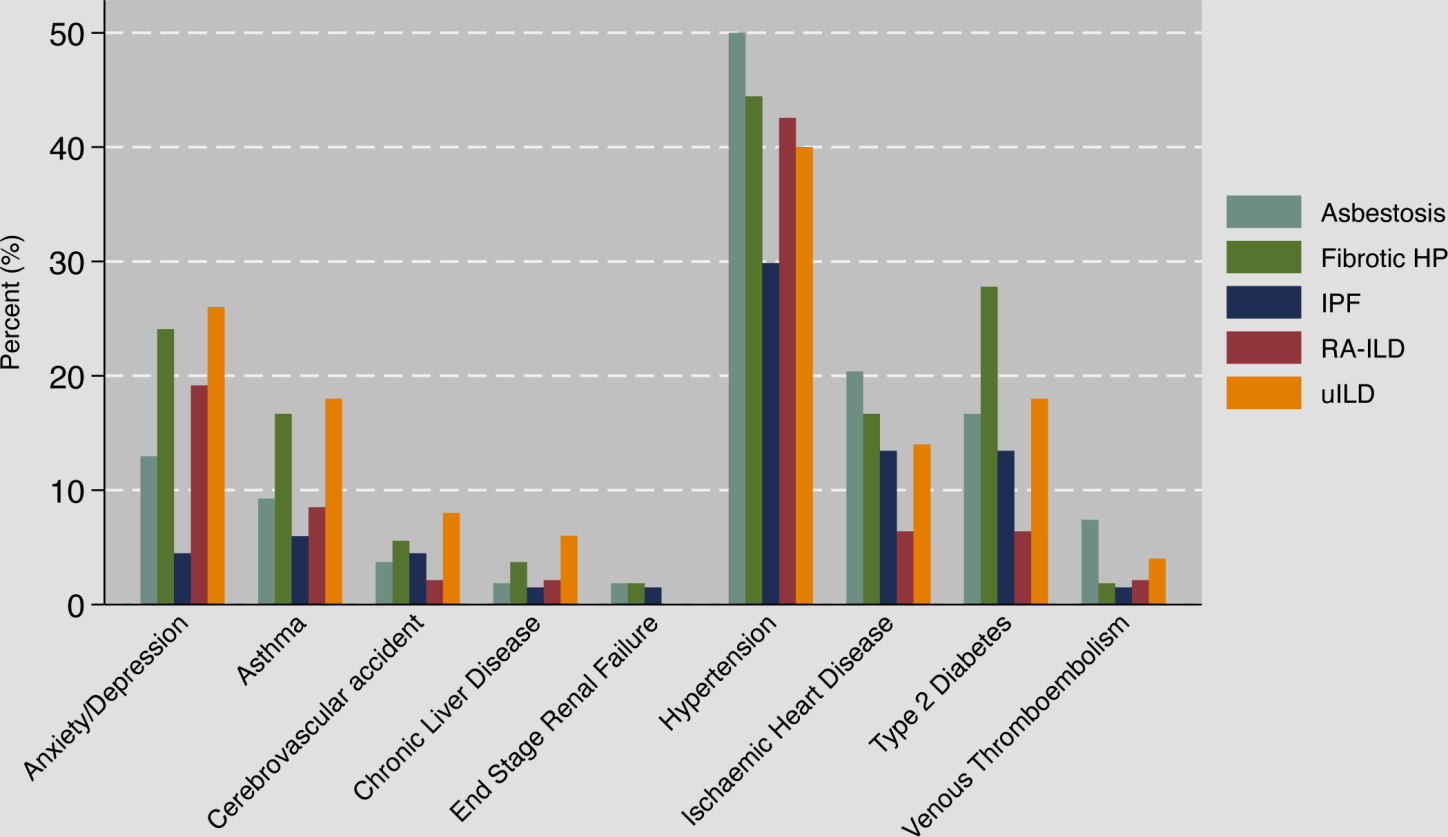
Baseline co-morbidities stratified by ILD subtypes. HP, hypersensitivity pneumonitis; ILD, interstitial lung disease; IPF, idiopathic pulmonary fibrosis; RA-ILD, rheumatoid arthritis ILD; uILD, unclassifiable ILD.

Variations in the prevalence of comorbidities were observed, highlighting the diverse clinical profiles within the study population. Systemic hypertension emerged as the most prevalent comorbidity (40.8% overall) affecting 29.9% of IPF and 44.4% of non-IPF participants (figure 1, online supplemental table). The prevalence of systemic hypertension across non-IPF subtypes ranged between 40% and 50% (p=0.23). Notable prevalence rates were also observed for type 2 diabetes mellitus (16.5% overall; 13.4% IPF, 17.6% non-IPF), ischaemic heart disease (14.3% overall; 13.4% IPF, 14.6% non-IPF) and asthma (11.4% overall; 6% IPF, 13.2% non-IPF). Lower rates were observed for venous thromboembolism (3.3% overall; 1.5% IPF, 3.9% non-IPF) and a previous history of cerebrovascular accident (4.8% overall; 4.5% IPF, 4.9% non-IPF). Anxiety and depression were reported in 16.9% of participants (4.5% IPF, 21% non-IPF), suggesting potential associations between ILD subtype and mental health comorbidities. Within the non-IPF subtypes, the highest occurrence of anxiety and depression was in the uILD group (28%).

Treatments with protein pump inhibitors (54.4%), statins (52.6%) and anticoagulation (24.6%) were common, with no significant difference between IPF and non-IPF groups (online supplemental table). Among non-IPF participants (n=205), 103 individuals (50.2%) were not receiving any disease-specific treatment at baseline. A larger proportion of participants with non-IPF were receiving either corticosteroid or immunomodulatory therapy (43.9%) when compared with IPF subjects (9%). The difference was largely driven by immunosuppressant use among participants with fibrotic HP (53.7%) and RA-ILD (80.9%). In contrast, only 11.1% of individuals with asbestosis were receiving immunosuppressive therapy. Antifibrotics were prescribed to 13.6% of participants, with a higher prevalence in IPF (26.9%) compared with the non-IPF (9.3%) group (p<0.001).

### Bloods

No significant differences were observed in alanine aminotransferase (ALT), albumin, calcium, haemoglobin levels or white cell counts (WCC) between IPF and non-IPF groups (table 2). Lower WCCs and neutrophil counts were observed in individuals with asbestosis compared with other ILD subtypes (p=0.02 and p=0.005, respectively). Platelet counts were comparable between IPF (246.2±61.8) and non-IPF groups (257·1±70.2). Significantly elevated platelet counts were observed in RA-ILD patients relative to other ILD subtypes (p=0.008), indicating potential inflammatory activity. Eosinophil, monocyte and lymphocyte counts did not significantly differ between IPF and non-IPF groups or across the different ILD subtypes. In terms of autoimmune markers, there were no significant differences in the prevalence of antinuclear antibodies, perinuclear antineutrophil cytoplasmic antibodies, cytoplasmic antineutrophil cytoplasmic antibodies, rheumatoid factor (RhF) and anti-cyclic citrullinated peptide (anti-CCP) antibodies between IPF and non-IPF groups. Within ILD subtypes, RhF was markedly more prevalent in RA-ILD, with 61.7% testing positive compared with much lower percentages in other subtypes (p<0.001). Anti-CCP antibodies showed a similar pattern with 36.2% positivity in RA-ILD, significantly higher than other ILD subtypes (p<0.001).

**Table 2:** Baseline laboratory and immunological characteristics

	All (n=272)	IPF (n=67)	Non-IPF (n=205)	Asbestosis (n=54)	Fibrotic HP (n=54)	RA-ILD (n=47)	uILD (n=50)
ALT (U/L)	22.9 (11)	24.0 (9.9)	22.6 (11.3)	20.7 (8.1)	23.0 (12.1)	20.7 (10.7)	25.8 (12.8)
Albumin (g/L)	39.6 (4.5)	40.2 (3.4)	39.5 (4.8)	40.3 (4.4)	39.7 (3.9)	37.8 (3.9)	40.0 (6.3)
Calcium (mmol/L)	2.36 (0.1)	2.34 (0.08)	2.36 (0.1)	2.36 (0.09)	2.33 (0.1)	2.35 (0.09)	2.40 (0.11)
Haemoglobin (g/L)	121.9 (45.3)	128.3 (43.8)	120 (45.7)	117.2 (49.4)	131.6 (32.4)	107.9 (49.8)	121.7 (48.2)
White cell count (10^9^ /L)	8.4 (2.5)	8.4 (2.2)	8.4 (2.6)	7.7 (2.4)	9.2 (2.7)	8.8 (2.3)	8.0 (2.7)
Platelets (10^9^ /L)	254.5 (68.4)	246.2 (61.8)	257.1 (70.2)	240.4 (61.4)	256.8 (54.3)	286.2 (91.3)	247.4 (64.8)
Neutrophils (10^9^ /L)	5.68 (2.43)	5.42 (2.01)	5.76 (2.55)	4.96 (2.1)	6.44 (2.75)	6.32 (2.78)	5.33 (2.24)
Eosinophils (10^9^ /L)	0.27 (0.26)	0.29 (0.19)	0.26 (0.27)	0.31 (0.36)	0.24 (0.23)	0.23 (0.26)	0.25 (0.21)
Monocytes (10^9^ /L)	0.66 (0.27)	0.69 (0.28)	0.65 (0.26)	0.68 (0.27)	0.66 (024)	0.66 (0.25)	0.61 (0.28)
Lymphocytes (10^9^ /L)	1.78 (0.78)	1.96 (0.83)	1.73 (0.75)	1.66 (0.59)	1.77 (0.81)	1.71 (0.75)	1.78 (0.86)
RhF							
Positive	42 (15.4%)	3 (4.5%)	39 (19%)	1 (1.9%)	4 (7.4%)	29 (61.7%)	5 (10%)
Negative	79 (29%)	27 (40.3%)	52 (25.4%)	12 (22.2%)	21 (38.9%)	3 (6.4%)	16 (32%)
Not checked/unavailable	151 (55.5%)	37 (55.2%)	114 (55.6%)	41 (75.9%)	29 (53.7%)	15 (31.9%)	29 (58%)
Anti-CCP							
Positive	23 (8.5%)	2 (3%)	21 (10.2%)	0	2 (3.7%)	17 (36.2%)	2 (4%)
Equivocal	4 (1.5%)	1 (1.5%)	3 (1.5%)	0	0	3 (6.4%)	0
Negative	73 (26.8%)	24 (35.8%)	49 (23.9%)	11 (20.4%)	16 (29.6%)	6 (12.8%)	16 (32%)
Not checked/unavailable	172 (63.2%)	40 (59.7%)	132 (64.4%	43 (79.6%)	36 (66.7%)	21 (44.7%)	32 (64%)
ANA							
Positive	41 (15.1%)	9 (13.4%)	32 (15.6%)	2 (3.7%)	9 (16.7%)	12 (25.5%)	9 (18%)
Negative	92 (33.8%)	26 (38.8%)	66 (32.2%)	13 (24.1%)	18 (33.3%)	13 (27.7%)	22 (44%)
Not checked/unavailable	139 (51.1%)	32 (47.8%)	107 (52.2%)	39 (72.2%)	27 (50%)	22 (46.8%)	19 (38%)
pANCA							
Positive	20 (7.4%)	3 (4.5%)	17 (8.3%)	1 (1.9%)	7 (13%)	3 (6.4%)	6 (12%)
Negative	48 (17.7%)	11 (16.4%)	37 (18.1%)	4 (7.4%)	15 (27.8%)	5 (10.6%)	13 (26%)
Not checked/unavailable	204 (75%)	53 (79.1%)	151 (73.7%)	49 (90.7%)	32 (59.3%)	39 (83%)	31 (62%)
cANCA							
Positive	12 (4.4%)	3 (4.5%)	9 (4.4%)	0	3 (5.6%)	2 (4.3%)	4 (8%)
Negative	74 (27.2%)	16 (23.9%)	58 (28.3%)	8 (14.8%)	22 (40.7%)	7 (14.9%)	21 (42%)
Not checked/unavailable	186 (68.4%)	48 (71.6%)	138 (67.3%)	46 (85.2%)	29 (53.7%)	38 (80.9%)	25 (50%)

This table displays the baseline laboratory and immunological characteristics of participants with different subtypes of ILD. Data are presented as proportions and percentages or mean and SD.

ALT, alanine aminotransferase (U/L); ANA, anti-nuclear antibody; anti-CCP, anti-cyclic citrullinated peptide; cANCA, cytoplasmic anti-neutrophil cytoplasmic antibodies; HP, hypersensitivity pneumonitis; ILD, interstitial lung disease; IPF, idiopathic pulmonary fibrosis; pANCA, perinuclear anti-neutrophil cytoplasmic antibodies; RA-ILD, rheumatoid arthritis ILD; RhF, rheumatoid factor; uILD, unclassifiable ILD.

### Exposures

Previous occupational exposure was reported in 68.8% of participants (64.2% IPF, 70.2% non-IPF) (table 3). Asbestos exposure was the most prevalent, affecting 36% of the overall cohort (22.4% IPF, 40.5% non-IPF). Among non-asbestosis participants, 20.2% reported asbestos exposure, with significant exposure reported in 9.2%. Exposure to other agents was also common in the overall cohort, including mineral dust (25.4%), metal dust (22.1%), welding fumes (20.2%) and wood dust (19.5%). Bird exposure history was reported by 40.4% of all participants, with 41.8% of IPF and 40% of non-IPF reporting such exposure. There were no significant differences in bird exposure within the ILD subtypes (p=0.35). Among the cohort reporting bird exposure, budgies were the most frequently implicated (60%), followed by parrots (10.9%) and canaries (7.3%).

**Table 3:** Baseline occupational and environmental exposures

	All (n=272)	IPF (n=67)	Non-IPF (n=205)	Asbestosis (n=54)	Fibrotic HP (n=54)	RA-ILD (n=47)	uILD (n=50)
Acids (industrial)	30 (11%)	9 (13.4%)	21 (10.2%)	10 (18.5%)	5 (9.3%)	3 (6.4%)	3 (6%)
Asbestos	98 (36%)	15 (22.4%)	83 (40.5%)	54 (100%)	9 (16.7%)	10 (21.3%)	10 (20%)
Bird	110 (40.4%)	28 (41.8%)	82 (40%)	22 (40.7%)	24 (44.4%)	22 (46.8%)	14 (28%)
Cutting oils/fluids	37 (13.6%)	12 (17.9%)	25 (12.2%)	13 (24.1%)	6 (11.1%)	3 (6.4%)	3 (6%)
Farm animals	20 (7.4%)	1 (1.5%)	19 (9.3%)	3 (5.6%)	9 (16.7%)	2 (4.3%)	5 (10%)
Grain dust	23 (8.5%)	7 (10.5%)	16 (7.8%)	8 (14.8%)	5 (9.3%)	2 (4.3%)	1 (2%)
Hairdressing products	6 (2.2%)	1 (1.5%)	5 (2.4%)	0	3 (5.6%)	1 (2.1%)	1 (2%)
Metal dust	60 (22.1%)	20 (29.9%)	40 (19.5%)	16 (29.6%)	10 (18.5%)	7 (14.9%)	7 (14%)
Mineral dust	69 (25.4%)	13 (19.4%)	56 (27.3%)	29 (53.7%)	9 (16.7%)	8 (17%)	10 (20%)
Paint	46 (16.9%)	7 (10.5%)	39 (19%)	17 (31.5%)	8 (14.8%)	4 (8.5%)	10 (20%)
Paper dust	30 (11%)	9 (13.4%)	21 (10.2%)	7 (13%)	8 (14.8%)	3 (6.4%)	3 (6%)
Textile dust	33 (12.1%)	8 (11.9%)	25 (12.2%)	12 (22.2%)	6 (11.1%)	6 (12.8%)	1 (2%)
Welding fumes	55 (20.2%)	15 (22.4%)	40 (19.5%)	18 (33.3%)	11 (20.4%)	6 (12.8%)	5 (10%)
Wood dust	53 (19.5%)	5 (7.5%)	48 (23.4%)	28 (51.9%)	7 (13%)	3 (6.4%)	10 (20%)

This table presents the distribution of various occupational and environmental exposures reported by participants with different subtypes of ILD. Data are presented as percentages.

HP, hypersensitivity pneumonitis; ILD, interstitial lung disease; IPF, idiopathic pulmonary fibrosis; RA-ILD, rheumatoid arthritis ILD; uILD, unclassifiable ILD.

### Quality of life scores

Participants reported a significant impact on HRQOL across various instruments (table 4). The median IPARC distress score was 9.5 (IQR 5–10), with no difference observed between IPF and non-IPF groups (p=0.07). The mean overall KBILD score was 57.1±14.6, with lower scores in the activity domain (44·7±21.3), compared with the breathlessness (65·1±24.9) and psychological domains (58·9±19.8). These observations were consistent across both IPF and non-IPF groups. The median MRC score was 2 (IQR 2–3) indicating significant breathlessness on exertion, with no variation between IPF and non-IPF (p=0.17). The median EQ-5D-5L index was 0.8 (IQR 0.66–0.92), indicating relatively poorer health states among non-IPF individuals (0.79, IQR 0.62–0.90), compared with those with IPF (0.83, IQR: 0.73–0.94) (p=0.03). The median visual analogue scale score was 70 (IQR: 50–85) with no difference observed between IPF and non-IPF (p=0.25). The overall LCQ score was 17.0 (IQR 13.2–19.4), with no discernible variation between IPF and non-IPF groups. However, significant divergence was observed in the physical domain across ILD subtypes (p=0.04) with lower scores recorded in asbestosis (5.1, IQR: 3.9–5.9) and higher scores noted in RA-ILD (5.8, IQR: 4.9–6.5).

**Table 4:** Baseline quality of life scores

	All (n=272)	IPF (n=67)	Non-IPF (n=205)	Asbestosis (n=54)	Fibrotic HP (n=54)	RA-ILD (n=47)	uILD (n=50)
IPARC	9.5 (5-10)	9 (4-14)	10 (5–19)	10 (6–18)	10 (5–19)	9 (3-20)	9.5 (5-20)
KBILD							
Total	57.1 (14.6)	58.2 (10.3)	56.7 (15.8)	54.6 (14.6)	55.6 (12.4)	60.2 (17.7)	56.8 (18.0)
Breathlessness domain	65.1 (24.9)	69.9 (22.5)	63.5 (25.4)	58.7 (25.3)	63.7 (24.5)	69.9 (26.5)	62.6 (24.9)
Activities domain	44.7 (21.3)	47.2 (18.2)	43.8 (22.2)	42.9 (19.7)	41.6 (18.7)	47.9 (22.9)	43.4 (27.3)
Psychological domain	58.9 (19.8)	59.3 (16.7)	58.8 (20.8)	56.6 (20.0)	58.5 (18.8)	62.9 (22.9)	57.4 (21.6)
EQ-5D-5L							
Health score	70 (50–85)	75 (60–85)	70 (50–80)	70 (50–80)	70 (50–85)	71 (50–85)	65 (50–90)
EQ-5D index	0.80 (0.66–0.92)	0.83 (0.73–0.94)	0.79 (0.62–0.90)	0.79 (0.66–0.88)	0.81 (0.63–0.94)	0.75 (0.57–0.89)	0.75 (0.49–0.94)
MRC							
Score	2 (2-3)	2 (2-3)	2 (2-3)	3 (2-3)	2.5 (2-3)	2 (2-3)	2 (2-3)
1	35 (13%)	8 (12.3%)	27 (13.2%)	7 (13%)	4 (7.4%)	8 (17%)	8 (16.3%)
2	116 (43.1%)	34 (52.3%)	82 (40.2%)	19 (35.2%)	23 (42.6%)	22 (46.8%)	18 (36.7%)
3	70 (26%)	15 (23.1%)	55 (27%)	16 (29.6%)	18 (33.3%)	8 (17%)	13 (26.5%)
4	38 (14.1%)	7 (10.8%)	33 (15.2%)	11 (20.4%)	7 (13.0%)	7 (14.9%)	6 (12.2%)
5	10 (3.7%)	1 (1.5%)	9 (4.4%)	1 (1.9%)	2 (3.7%)	2 (4.3%)	4 (8.2%)
LCQ							
Total	17.0 (13.2–19.4)	17.4 (14.2–19.3)	16.7 (12.9–19.5)	15.9 (12.1–18.5)	16.5 (13.1–18.7)	18.8 (13.9–20.1)	16.1 (11.8–19.5)
Physical	5.5 (4.38–6.13)	5.5 (4.63–6.38)	5.5 (4.13–6.13)	5.1 (3.9–5.9)	5.4 (4.1–6.3)	5.8 (4.9–6.5)	5.3 (4.0–6.0)
Psychological	5.86 (4.29–6.71)	5.86 (4.57–6.57)	5.71 (4.14–6.71)	5 (4.0–6.6)	5.4 (4.4–6.4)	6.4 (4.7–6.9)	5.9 (4.0–6.7)
Social	5.75 (4.5–6.75)	5.75 (5.0–6.75)	5.75 (4.5–6.75)	5.8 (4.5–6.8)	5.6 (4.5–6.5)	6.5 (4.5–7)	5.5 (4.3–6.8)

This table compares the various quality of life scores among participants with different subtypes of ILD. Data are presented as means and SD, medians and IQRs, or percentages.

HP, hypersensitivity pneumonitis; ILD, interstitial lung disease; IPF, idiopathic pulmonary fibrosis; MRC, Medical Research Council; RA-ILD, rheumatoid arthritis ILD; uILD, unclassifiable ILD.

## Discussion

The INJUSTIS study aims to identify genetic, proteomic and clinical biomarkers that can predict progressive fibrotic phenotypes from more stable ones, regardless of aetiology. This comprehensive analysis of baseline demographic and clinical characteristics from recruited participants offers valuable insights that deepen our understanding of the nuanced similarities and differences across fibrotic ILD subtypes.

The participant demographic consistently comprised predominantly older white males with a history of smoking, aligning with established knowledge about common ILD risk factors.^20^ Male predominance was less pronounced in non-IPF conditions, suggesting potential differences in aetiology or exposure histories. The exception was in asbestosis, which was almost exclusively male, underscoring the occupational link to traditionally male-dominated industries such as construction and shipbuilding, where asbestos exposure is prevalent.^21^ Similarities in family history were reported across ILD subtypes, highlighting the possibility of shared genetic factors that could influence susceptibility or disease progression.^22^ Similarities in the impairment of baseline pulmonary function, specifically FVC and DL,_CO,_ were observed across ILD subtypes, suggesting comparable severity at diagnosis, despite possible differences in aetiological factors. The relatively low proportion of IPF participants on antifibrotic therapy likely reflects early-stage disease at the point of recruitment, the short interval between diagnosis and enrolment and evolving access to antifibrotics over the recruitment period, which overlapped with updates to National Institute for Health and Care Excellence guidelines and prescribing practices.

Widespread presence of comorbid conditions including hypertension, ischaemic heart disease and diabetes was observed. While these findings may reflect the older, predominantly male demographic with a smoking history, further research is needed to explore the potential links between systemic inflammation, fibrosis and impacts on multiple organ systems.^23^ The systemic nature of these comorbidities highlights their potential role as risk factors for the development or progression of ILD. Biomarkers that can delineate the impact of such systemic diseases on ILD progression could be invaluable and may include markers of systemic inflammation, oxidative stress or other metabolic syndromes that exacerbate fibrotic pathways.^24 25^ These findings reiterate the importance of holistic care strategies and emphasise the need for complete cardiovascular and metabolic management alongside respiratory care.

Individuals with uILD exhibited higher rates of anxiety and depression compared with other ILD subtypes. In contrast, the lowest prevalence of anxiety and depression was observed in the IPF group. The inherent uncertainty and variability associated with a uILD diagnosis may lead to greater psychological stress and a diminished sense of control over the disease process. In IPF, the availability of more structured diagnostic and treatment pathways, alongside better-established support networks and greater understanding of natural history, likely provides patients with a clearer understanding of their condition and more effective psychological coping mechanisms. These findings indicate that psychosocial factors and mental health support need to be tailored specifically for different ILD subtypes, acknowledging that patient experiences and mental health needs can vary significantly depending on the nature and prognosis of the underlying disease. Despite the severity and variability of these diseases, HRQOL scores were similar across all subtypes, reinforcing the substantial impact of ILD on daily living and overall well-being. This observation reinforces the importance of incorporating quality of life measurements into routine clinical assessments and trials, ensuring that treatment plans and novel therapies address both the physical and emotional aspects of ILD.

The similarities in bird exposure across different ILD subtypes prompt a critical re-evaluation of how bird-related exposures are interpreted in the diagnostic process, particularly in fibrotic HP, where such exposures have traditionally been viewed as primary causative factors.^26 27^ This observation suggests that some diagnostic criteria may rely on anecdotal or unsubstantiated links between exposure and disease, which could lead to misclassification or overemphasis on certain environmental factors.^28^ Our findings suggest that bird exposure may represent a generalised environmental risk factor that could predispose or exacerbate underlying pulmonary conditions. This highlights the need for further research into gene-environment interactions in ILD as well as the importance of detailed environmental assessments and the development of biomarkers that can differentiate between aetiological factors.

This baseline analysis provides a foundation for further research on ILD biomarkers and disease mechanisms that could identify common fibrogenic pathways across ILD aetiologies. Future biomarker studies should aim to dissect the complex interactions between genetic susceptibility, environmental exposures and clinical manifestations of ILD. This integration of clinical, environmental and psychosocial factors should enable the development of predictive models of disease progression, response to therapy and overall patient outcomes. This multifaceted approach will foster personalised medicine strategies that effectively address the complex nature of ILD, enhancing prognostication and treatment for patients across various ILD subtypes. By addressing these elements comprehensively, future research can transcend traditional diagnostic boundaries, offering new insights into the aetiology of ILDs and fostering the development of innovative treatment paradigms.

The INJUSTIS study has several strengths, notably its prospective, multicentre design across 24 UK sites, providing a comprehensive baseline evaluation that includes detailed demographic, clinical and exposure histories, alongside extensive physiological and quality of life assessments. These elements contribute to a robust dataset that facilitates nuanced analyses of disease characteristics.

However, this study has limitations. The cohort predominantly consists of older white males with a history of smoking, which may limit the applicability of the findings to more diverse populations, including women and individuals from different ethnic backgrounds. The overall sample size was designed to distinguish between progressive and stable fibrotic ILD over the follow-up period and is not sufficient for granular ILD subtype comparisons. However, notable differences consistent with underlying diagnoses were observed (eg, treatment in non-IPF ILD; immunology in RA-ILD and exposures in asbestosis) alongside a surprising absence of differences in avian exposure. The median time from diagnosis to recruitment was under 6 months. Despite this, the IPF group was generally milder than other cohorts.^29^ Recruitment delays due to the COVID-19 pandemic led to potential survivor bias. In addition, the recruitment period was extended due to the COVID-19 pandemic, potentially introducing temporal biases. For instance, changes in treatment protocols and patient management practices over the extended recruitment period could affect the comparability of data collected at different times. Additionally, the reliance on patient-reported exposure histories and family history is subjected to recall bias, which may not accurately reflect environmental or inherited risk. Finally, the study faced challenges in recruiting equal numbers of participants across all ILD subtypes, particularly in the IPF group where recruitment was completed earlier. However, we mitigated this by over-recruiting in this group until the other subtypes achieved their target recruitment.

In conclusion, this baseline study provides a comprehensive evaluation of a fibrotic ILD cohort, offering insights into the similarities and distinctions across ILD subtypes. The demographic, clinical and exposure data, coupled with longitudinal follow-up, establish a robust foundation for future planned research integrating advanced genomic, transcriptomic and proteomic analyses, with a vision to support personalised treatment strategies in PPF.

## Supplementary material

10.1136/bmjresp-2024-003112 online supplemental file 1

## Data Availability

Data are available upon reasonable request.
