# Supplementary material for: Comprehensive characterisation of individuals with fibrotic interstitial lung disease: baseline insights from the INJUSTIS study
Source: BMJ Open Respir Res. 2026 Jan 20;13(1):e003112. doi: 10.1136/bmjresp-2024-003112 (PMC12820847; doi:10.1136/bmjresp-2024-003112)
Supplement: online supplemental file 1 [file bmjresp-13-1-s001.docx]

Supplementary material

Data collection form Pages 2-5

Supplemental Table 1 Page 6

| Is the participant CURRENTLY being treated for: | |
| --- | --- |
| **YES NO**  Hypertension:  Ischaemic heart disease:  Stroke:  Diabetes:  Blood clot (DVT or PE):  Cancer: | **YES NO**  Liver disease:  Renal failure:  Asthma:  Rheumatoid Arthritis:  Depression/Anxiety:  Osteoarthritis: |

| **Family history of Pulmonary Fibrosis:** |
| --- |
| **YES NO YES NO YES NO**  Family 1^st^ degree 2^nd^ degree  history: relatives: relatives:  1^st^ degree relatives include parents, offspring and siblings (share 50% of their genes with individuals)  2^nd^ degree relatives include grandparents, grandchildren, aunts, uncles, nephews, nieces of half-siblings |

| **Smoking History** |
| --- |
| CURRENT If current/ex, how many cigarettes did/do they smoke  per day on average?  EX  If current/ex how many years have/did they smoke for?  NEVER  **PACK YEARS**  Pack year is calculated by multiplying the number of **packs** of cigarettes smoked per day (20 in a pack) by the number of **years** smoked for |

| **Occupational History** | | | | | |
| --- | --- | --- | --- | --- | --- |
|  | | |  | | |
| 1. Manager/Senior Official 2. Professional 3. Associate professional/technical 4. Administrative/secretarial 5. Skilled Trades | | | 1. Personal services 2. Sales/Customer Services 3. Process, Plant and Machine operatives 4. Elementary Occupations | | |
| Please list ALL of the jobs that the participant has done and code as above: | | | | | |
| Code | Job Title | What did they do/what service did they provide | | Year started | Year finished |
|  |  |  | |  |  |
|  |  |  | |  |  |
|  |  |  | |  |  |
|  |  |  | |  |  |
|  |  |  | |  |  |
|  |  |  | |  |  |
|  |  |  | |  |  |
|  |  |  | |  |  |
|  |  |  | |  |  |
|  |  |  | |  |  |
|  |  |  | |  |  |
|  |  |  | |  |  |
|  |  |  | |  |  |
|  |  |  | |  |  |
|  |  |  | |  |  |
|  |  |  | |  |  |
|  |  |  | |  |  |
|  |  |  | |  |  |

| **Occupational Exposures** | | |
| --- | --- | --- |
| During the participants working life, were they ever REGULARLY exposed to any of the following: | | |
| Asbestos  (qualify further below)  Mineral or stone dust  Paper dust  Metal dust  Welding fumes | Cutting oils or fluids  Grain dust  Strong acids  Wood dust  Textile dust | Hairdressing products  Paint  Farm animals |
|  | | |
| **Asbestos exposure** | | |
| If asbestos exposure:  Significant exposure: (Defined as at least 1 year of heavy exposure (manufacture of asbestos products, asbestos spraying, insulation work with asbestos materials, demolition of old buildings) or 5-10 years of moderate asbestos exposure (construction, shipbuilding etc.)  Yes No | | |

| **Bird history** | | |
| --- | --- | --- |
| Have they ever kept a bird: If yes, fill in below table  Yes No | | |
| Type of bird | Date from bird kept | Date until bird kept |
|  |  |  |
|  |  |  |
|  |  |  |
|  |  |  |
|  |  |  |

| **Medications** | |
| --- | --- |
| If the participant is taking the following medications, please record and code below, and record all other medications too: | |
| 1. Pirfenidone 2. Nintedanib 3. Immunosuppressants/immunomodulators 4. Steroids 5. Long term antibiotics | 1. PPI 2. H2RA 3. Statins 4. Chemotherapy 5. Anticoagulants |

| **Code** | **Drug name** | **Date commenced** | **> 3 months** | | **Ongoing at end of study** | | **Date finished**  **dd/mm/yyyy** |
| --- | --- | --- | --- | --- | --- | --- | --- |
|  |  |  | **Yes** | **No** | **Yes** | **No** |  |
|  |  |  |  |  |  |  |  |
|  |  |  |  |  |  |  |  |
|  |  |  |  |  |  |  |  |
|  |  |  |  |  |  |  |  |
|  |  |  |  |  |  |  |  |
|  |  |  |  |  |  |  |  |
|  |  |  |  |  |  |  |  |
|  |  |  |  |  |  |  |  |
|  |  |  |  |  |  |  |  |
|  |  |  |  |  |  |  |  |
|  |  |  |  |  |  |  |  |
|  |  |  |  |  |  |  |  |
|  |  |  |  |  |  |  |  |
|  |  |  |  |  |  |  |  |
|  |  |  |  |  |  |  |  |
|  |  |  |  |  |  |  |  |
|  |  |  |  |  |  |  |  |
|  |  |  |  |  |  |  |  |
|  |  |  |  |  |  |  |  |
|  |  |  |  |  |  |  |  |
|  |  |  |  |  |  |  |  |

|  | **All (N=272)** | **IPF (n=67)** | **Non-IPF (n=205)** | **Asbestosis (n=54)** | **Fibrotic HP (n=54)** | **RA-ILD (n=47)** | **uILD (n=50)** |
| --- | --- | --- | --- | --- | --- | --- | --- |
| **Co-morbidities** |  |  |  |  |  |  |  |
|  |  |  |  |  |  |  |  |
| Asthma | 31 (11.4%) | 4 (6%) | 27 (13.2%) | 5 (9.3%) | 9 (16.7%) | 4 (8.5%) | 9 (18%) |
| Hypertension | 111 (40.8%) | 20 (29.9%) | 91 (44.4%) | 27 (50%) | 24 (44.4%) | 20 (42.6%) | 20 (40%) |
| Type 2 Diabetes Mellitus | 45 (16.5%) | 9 (13.4%) | 36 (17.6%) | 9 (16.7%) | 15 (27.8%) | 3 (6.4%) | 9 (18%) |
| Ischaemic Heart Disease | 39 (14.3%) | 9(13.4%) | 30 (14.6%) | 11 (20.4%) | 9 (16.7%) | 3 (6.4%) | 7 (14%) |
| Anxiety/Depression | 46 (16.9%) | 3 (4.5%) | 43 (21%) | 7 (13%) | 13 (24.1%) | 9 (19.2%) | 14 (28%) |
| Venous thromboembolism | 9 (3.3%) | 1 (1.5%) | 8 (3.9%) | 4 (7.4%) | 1 (1.9%) | 1 (2.1%) | 2 (4%) |
| Cerebrovascular accident | 13 (4.8%) | 3 (4.5%) | 10 (4.9%) | 2 (3.7%) | 3 (5.6%) | 1 (2.1%) | 4 (8%) |
|  |  |  |  |  |  |  |  |
| **Medications** |  |  |  |  |  |  |  |
|  |  |  |  |  |  |  |  |
| Anticoagulation | 67 (24.6%) | 12 (17.9%) | 55 (26.8%) | 19 (35.2%) | 16 (29.6%) | 11 (23.4%) | 9 (18%) |
| Anti-fibrotics | 37 (13.6%) | 18 (26.9%) | 19 (9.3%) | 7 (13%) | 3 (5.6%) | 4 (8.5%) | 5 (10%) |
| H2 receptor antagonists | 14 (5.2%) | 1 (1.5%) | 13 (6.3%) | 4 (7.4%) | 3 (5.6%) | 3 (6.4%) | 3 (6%) |
| Long term antibiotics | 21 (7.7%) | 1 (1.5%) | 20 (9.8%) | 4 (7.4%) | 5 (9.3%) | 4 (8.5%) | 7 (14%) |
| Proton Pump Inhibitors | 148 (54.4%) | 30 (44.8%) | 118 (57.6%) | 31 (57.4%) | 33 (61.1%) | 32 (68.1%) | 22 (44%) |
| Statins | 143 (52.6%) | 33 (49.3%) | 110 (53.7%) | 38 (70.4%) | 27 (50%) | 21 (44.7%) | 24 (48%) |
| Steroids or Immunomodulatories | 96 (35.3%) | 6 (9%) | 90 (43.9%) | 6 (11.1%) | 29 (53.7%) | 38 (80.9%) | 17 (34%) |

Supplemental Table 1 – Baseline co-morbidities and medications of included participants. Data are presented as percentages.
